# The systemic inflammatory response index is associated with chronic kidney disease in patients with hypertension: data from the national health and nutrition examination study 1999–2018

**DOI:** 10.1080/0886022X.2024.2396459

**Published:** 2024-09-23

**Authors:** Yani Wang, Lihua Liao, Qian Guo, Ying Liao, Xueqin Lin, Huilan Li, Lin Deng, Yufei Deng, Danni Guo, Kaihong Chen, Yong Fang

**Affiliations:** Department of Cardiovascular Medicine, Longyan First Affiliated Hospital of Fujian Medical University, Longyan, Fujian, China

**Keywords:** Hypertension, chronic kidney disease, systemic inflammatory response index (SIRI), NHANES

## Abstract

**Background:**

Studies have shown that in hypertensive patients, chronic kidney disease (CKD) is associated with a poor prognosis. Inflammation is a highly important factor in the progression of CKD. Detecting systemic inflammation and intervening promptly in patients with hypertension may help reduce the risk of CKD. The systemic inflammatory response index (SIRI) is a tool used to measure the systemic inflammatory response, but its relationship with CKD in patients with hypertension remains uncertain.

**Methods:**

We utilized data from the National Health and Nutrition Examination Survey (NHANES), which was conducted between 1999 and 2018. The analysis included a total of 20,243 participants, categorized into three groups based on SIRI tertiles. Logistic regression analysis and restricted cubic spline analysis were used to examine the relationship between the SIRI and CKD.

**Results:**

In patients with hypertension, there was a notable relationship between the SIRI and the odds of developing CKD. After full adjustment, there was a 31% greater likelihood of developing CKD associated with each incremental increase of 1 unit in the SIRI (OR: 1.31, 95% CI: 1.24–1.39, *p* < 0.001). The groups with greater SIRI values exhibited greater odds of developing CKD than did the T1 group (T2: OR: 1.20, 95% CI: 1.04–1.38, *p* = 0.015; T3: OR: 1.69, 95% CI: 1.47–1.94, *p* < 0.001).

**Conclusion:**

A high SIRI is associated with an increased risk of CKD in hypertensive patients. The greater the SIRI is, the greater the risk of CKD in hypertensive patients.

## Introduction

Chronic kidney disease (CKD) is one of the most common chronic diseases worldwide and has become a serious public health problem, resulting in substantial medical expenses [1]. CKD is highly prevalent among individuals with hypertension, accounting for approximately 31%-51% of that population [2]. Early screening and intervention may help reduce the prevalence of CKD through in hypertensive patients.

Inflammation is one of the causes of kidney damage in hypertensive patients [3,4]. During hypertension, potent vasoactive molecules such as endothelin-1 and aldosterone can activate the inflammasome, which affects the vasculature and kidney, leading to CKD [5]. A previous study also revealed an association between serum inflammatory biomarkers and chronic kidney disease in hypertensive patients [6].

The systemic inflammatory response index (SIRI) is regarded as a composite inflammation index that can reflect the state of chronic inflammation in humans and is strongly related to cancer, hyperuricemia, rheumatoid arthritis, and stroke [7–9]. Increasing attention has been given to the role of the SIRI in cardiovascular diseases (CVDs), and studies have shown that the SIRI can predict the prognosis of patients with hypertension and heart failure [10,11]. However, it is unclear whether the SIRI is associated with CKD in hypertensive patients.

Therefore, the purpose of this study was to investigate the relationship between the SIRI and CKD in patients with hypertension, and to offer insights that could help inform strategies for preventing CKD in hypertensive patients.

## Methods

### Study design and population

The National Health and Nutrition Examination Survey (NHANES) is a comprehensive study conducted by the National Center for Health Statistics (NCHS) with the purpose of assessing the overall health and nutritional status of the general population in the United States. This survey is designed to be representative of the entire nation and provides valuable insights into the health and dietary habits of individuals and households. This study used a stratified multistage sampling design and surveyed 5,000 individuals annually. All participants underwent physiological measurements and laboratory tests.

**Figure 1.**
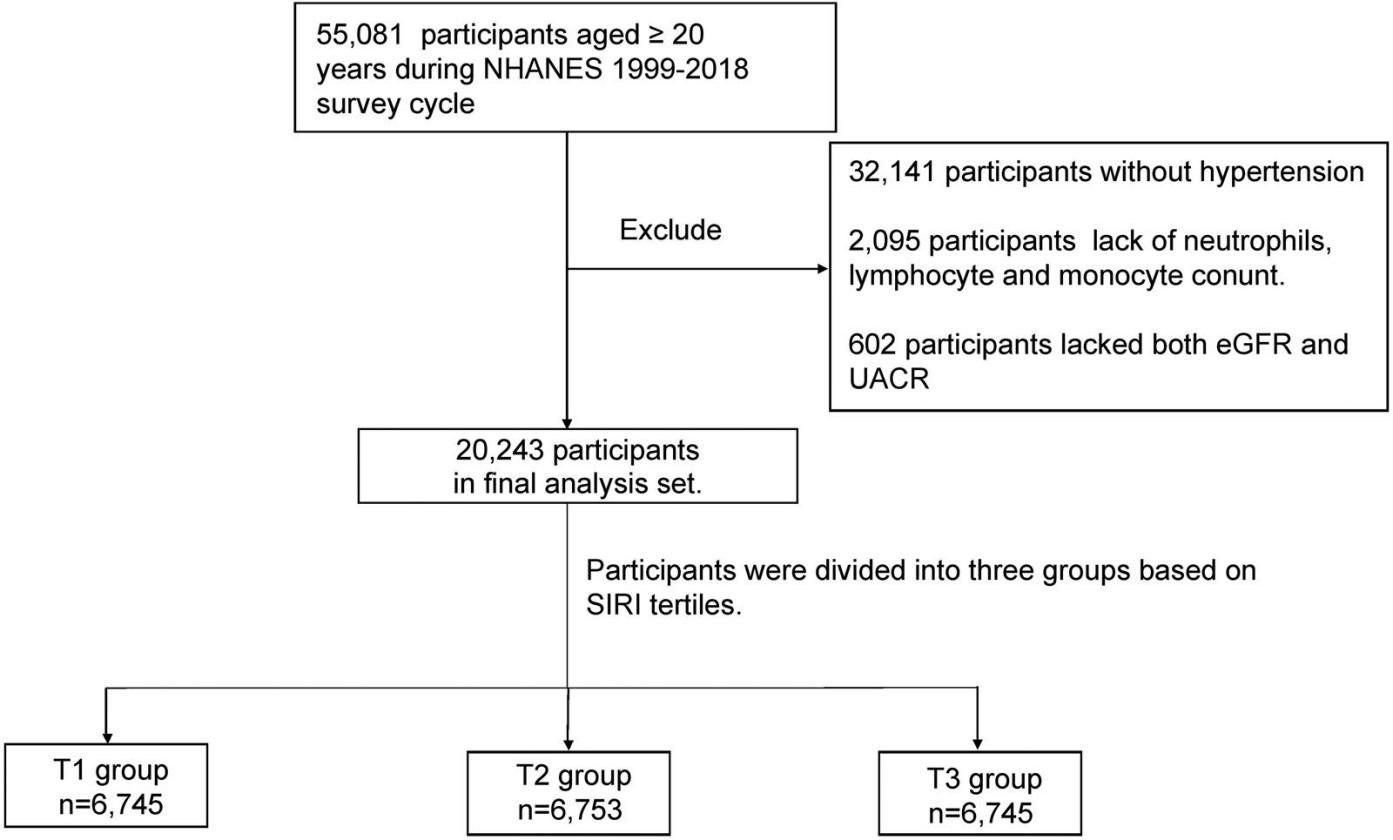
Flow chart of the study design.

The aim of this cross-sectional study was to determine the association between the SIRI and CKD in hypertensive patients from the NHANES in the US. As shown in Figure 1, a total of 55,081 individuals aged ≥ 20 years who participated in the NHANES during 1999–2018 were included in this study. Of these, 32,141 participants without hypertension were excluded. A total of 2,095 participants were excluded because of a lack of neutrophil, lymphocyte, or monocyte data. Additionally, 602 participants were excluded because of a lack of estimated glomerular filtration rate (eGFR) and urinary albumin/creatinine ratio (UACR) data. Ultimately, 20,243 participants were included in our study.

### Definition of hypertension

Participants were diagnosed with hypertension if they met any of the following criteria: 1. during the NHANES interview, responded ‘yes’ to the inquiry ‘Have you ever been informed by your doctor that you have hypertension?’; 2. average systolic blood pressure (SBP) or average diastolic blood pressure (DBP) exceeding 130 mmHg and 80 mmHg, respectively; or 3. self-reported use of medications that were prescribed for managing hypertension.

### Primary outcome

The main outcome was CKD. Participants were diagnosed with CKD if they presented with an eGFR below 60 mL/min/1.73 m^2^ calculated using the Chronic Kidney Disease Epidemiology Collaboration Equation (CKD-EPI) 2009 and/or a random UACR equal to or exceeding 30 mg/g [12].

### Calculation of the SIRI and the grouping method

The formula for calculating the SIRI is derived by multiplying the neutrophil count by the monocyte count and dividing it by the lymphocyte count. Participants were classified into three tertile groups based on their SIRI values: Group 1 (SIRI < 0.86), Group 2 (0.86≤ SIRI < 1.43), and Group 3 (SIRI ≥1.43). To verify the robustness of the results, regrouping was performed based on medians and quartiles.

### Assessment of covariates

Age, sex, race, education level, family-to-income ratio, alcohol consumption status, and smoking status were self-reported by the participants. Participants were diagnosed with diabetes mellitus (DM) if they met any of the following criteria: 1. previously told by a physician that they had DM; 2. hemoglobin A1c (HbA1c) greater than 6.5%; 3. fasting glucose ≥ 7.0 mmol/L; 4. random blood glucose ≥ 11.1 mmol/L; 5. two-hour oral glucose tolerance test (OGTT) blood glucose ≥ 11.1 mmol/L; or 6. use of diabetes medication or insulin. Dyslipidemia was considered to be indicated by total cholesterol ≥ 200 mg/dL, triglycerides ≥ 150 mg/dL, high-density lipoprotein ≤ 40 mg/dL for men and ≤ 50 mg/dL for women, or a low-density lipoprotein ≥ 130 mg/dL [13]. Additionally, people using antilipidemic medication were considered to have hyperlipidemia. CVD was diagnosed based on self-reported congestive heart failure, coronary heart disease, angina, heart attack, and stroke. Laboratory measurements, such as neutrophil counts, lymphocyte counts, monocyte counts, blood glucose, and blood lipids, were obtained from automated blood analysis equipment. Individuals whose body mass index (BMI) exceeded 30 were considered to have obesity. Covariates were selected based on their association with the outcomes as reported in previous studies [14–18].

### Statistical analyses

For continuous variables, we utilized the mean (standard error, SE) as the statistical measure, whereas proportions (with 95% confidence intervals, 95% CIs) were used for categorical variables. To compare the baseline characteristics between groups, we employed weighted χ2 tests to analyze categorical variables and utilized weighted analysis of variance (ANOVA) to analyze continuous variables.

The association between the SIRI and CKD was evaluated through logistic regression analyses. To improve the precision of the results, three models were formulated. Model 1 remained unadjusted, while Model 2 accounted for age, sex, and race. Model 3 was further adjusted for smoking status, alcohol consumption status, education level, family-to-income ratio, obesity, hyperlipidemia status, antihypertensive drugs use, DM status, and CVD status. A restricted cubic spline (RCS) regression analysis was performed to explore the potential nonlinear relationship between the SIRI and the risk of CKD. The adjustment variables included in the RCS analysis were the same as those included in Model 3. Furthermore, we investigated the relationship between the SIRI and CKD across various subgroups, including age, sex, obesity status, smoking status, alcohol consumption status, antihypertensive drugs use status, hyperlipidemia status, DM status and CVD status.

The data analyses were performed using the survey package in R software (version 4.0.4; R Foundation for Statistical Computing, Vienna, Austria). In all the analyses, a two-sided *P* value < 0.05 was considered to indicate statistical significance.

## Results

### Participant characteristics

Among the 20,243 eligible participants included in the study, the average age was 57.1 years (0.2), and nearly half of them (*n* = 9,968, 48.9%) were male. There were 4,183 participants (17.6%) with CVD, 6,006 (24.2%) with DM, 11,833 (55.3%) who were regularly taking antihypertensive drugs, and 6,538 (26.5%) with CKD. The participants were grouped into three categories based on SIRI tertiles: the T1 group (*n* = 6,745), T2 group (*n* = 6,753), and T3 group (*n* = 6,745). Among the three groups, there was considerable variation in the average age [T1: 54.8 (0.3) years vs. T2: 56.9 (0.3) years vs. T3: 59.4 (0.3) years, *p* < 0.001]. Moreover, individuals with higher SIRI values were more likely to be male [T1: 39.9% vs. T2: 48.3% vs. T3: 57.1%, *p* < 0.001] and were more likely to smoke [T1: 44.9% vs. T2: 49.9% vs. T3: 55.5%, *p* < 0.001]. Additionally, patients who had higher SIRI values were more likely to use antihypertensive drugs [T1: 50.6% vs. T2: 54.0% vs. T3: 60.5%, *p* < 0.001] and have CVD [T1: 13.0% vs. T2: 15.8% vs. T3: 23.4%, *p* < 0.001] and DM [T1: 21.5% vs. T2: 22.7% vs. T3: 28.1%, *p* < 0.001]. There was no observable between-group difference in alcohol consumption (*p* = 0.063). Further details can be found in Table 1.

**Table 1.** Baseline characteristics of the study population.

Characteristic	Overall *N* = 20243	T1 group *N* = 6745	T2 group *N* = 6753	T3 group *N* = 6745	*p* Value
SIRI	1.38 (0.01)	0.62 (0.00)	1.13 (0.00)	2.27 (0.01)	< 0.001
Age, years	57.13 (0.20)	54.75 (0.27)	56.85 (0.27)	59.41 (0.27)	< 0.001
Female, *n* (%)	10275 (51.10)	4012 (60.15)	3446 (51.67)	2817 (42.89)	< 0.001
Race, *n* (%)					< 0.001
Mexican American	2930 (5.70)	945 (6.14)	1103 (5.94)	882 (5.04)	
Non-Hispanic Black	4957 (12.90)	2647 (23.72)	1353 (9.95)	957 (6.69)	
Non-Hispanic White	9312 (70.90)	2001 (56.90)	3272 (73.58)	4039 (79.98)	
Other Hispanic	1512 (4.50)	519 (5.05)	546 (4.93)	447 (3.50)	
Other Race	1532 (6.10)	633 (8.19)	479 (5.60)	420 (4.78)	
Education level, *n* (%)					< 0.001
<12	6149 (30.42)	2129 (21.30)	2030 (18.62)	1990 (19.98)	
12	4950 (24.49)	1566 (24.37)	1638 (26.09)	1746 (27.95)	
>12	9116 (45.10)	3042 (54.33)	3074 (55.29)	3000 (52.07)	
PIR	2.956 (0.03)	2.940 (0.04)	3.021 (0.04)	2.904 (0.03)	0.022
Smoking status, *n* (%)	10092 (50.40)	2981 (44.87)	3320 (49.88)	3791 (55.46)	< 0.001
Alcohol consumption status, *n* (%)	11147 (67.70)	3676 (66.90)	3792 (69.21)	3679 (66.59)	0.063
BMI, kg/m^2^	30.77 (0.08)	30.27 (0.12)	30.95 (0.12)	31.03 (0.12)	< 0.001
Neutrophil, K/μL	4.45 (0.02)	3.13 (0.02)	4.26 (0.02)	5.75 (0.03)	< 0.001
Lymphocyte, K/μL	2.14 (0.02)	2.42 (0.04)	2.15 (0.01)	1.89 (0.01)	< 0.001
Monocyte, K/μL	0.59 (0.00)	0.46 (0.01)	0.57 (0.00)	0.72 (0.00)	< 0.001
TC, mg/dl	198.89 (0.54)	203.64 (0.82)	200.17 (0.74)	193.61 (0.84)	< 0.001
TG, mg/dl	170.62 (1.47)	158.97 (2.67)	177.37 (2.12)	173.70 (2.28)	< 0.001
LDL-C, mg/dl	115.55 (0.54)	120.39 (0.93)	116.11 (0.84)	110.18 (0.90)	< 0.001
HDL-C, mg/dl	52.31 (0.19)	54.19 (0.30)	51.74 (0.30)	51.28 (0.27)	< 0.001
FBS, mg/dl	106.68 (0.38)	105.20 (0.66)	105.25 (0.59)	109.36 (0.62)	< 0.001
eGFR, mL/min/1.73m^2^	84.19 (0.28)	88.54 (0.40)	84.60 (0.40)	80.11 (0.38)	< 0.001
UACR, mg/g	64.71 (3.18)	39.54 (3.60)	58.77 (4.88)	92.18 (7.39)	< 0.001
obesity, *n* (%)	9200 (46.42)	3019 (44.49)	3164 (49.20)	3017 (48.85)	< 0.001
HPL, *n* (%)	16593 (82.70)	5414 (80.84)	5624 (83.47)	5555 (83.53)	0.006
CVD, *n* (%)	4183 (17.60)	1014 (12.97)	1292 (15.80)	1877 (23.40)	< 0.001
DM, *n* (%)	6006 (24.20)	1868 (21.48)	1957 (22.73)	2181 (28.07)	< 0.001
Antihypertensive drug use, *n* (%)	11833 (55.30)	3664 (50.64)	3902 (54.04)	4267 (60.51)	< 0.001
CKD, *n* (%)	6538 (26.50)	1672 (20.18)	2058 (24.58)	2808 (33.81)	< 0.001

Values are, *n* (%) or mean (SE).

SIRI: systemic inflammatory response index; PIR: poverty income ratio; BMI: body mass index; TC: total cholesterol; TG: triglyceride; LDL-C: low-density lipoprotein cholesterol; HDL-C: high-density lipoprotein cholesterol; FBS: fasting blood glucose; eGFR: estimated glomerular filtration rate; UACR: urinary albumin/creatinine ratio; HPL: hyperlipidemia; CVD: cardiovascular disease; DM: diabetes mellitus; CKD: chronic kidney disease.

### The association between the SIRI and CKD

As shown in Table 1, those with higher SIRI values had a greater prevalence of CKD (T1 group:20.2% vs. T2 group:24.6% vs. T3 group: 33.8%). Univariate logistic regression analyses revealed a significant association between a 1-unit increase in the SIRI and a 44% increase in the likelihood of developing CKD (95% CI: 1.38–1.51; *p* < 0.001). The groups with higher SIRI values had a greater risk of CKD than did the T1 group (T2: OR: 1.29, 95% CI: 1.16–1.43, *p* < 0.001; T3: OR: 2.02, 95% CI: 1.83–2.23, *p* < 0.001). After full adjustment, the risk of developing CKD increased by 31% for every one-unit increase in the SIRI (95% CI: 1.24–1.39; *p* < 0.001). The risk of developing CKD was elevated in patients with a higher SIRI (T2: OR: 1.20, 95% CI: 1.04–1.38, *p* = 0.015; T3: OR: 1.69, 95% CI: 1.47–1.94, *p* < 0.001) (Table 2). The results did not change significantly after regrouping patients according to the median and quartiles. The group with a higher SIRI still had a greater risk of CKD (Supplementary Table 1).

**Table 2.** The association between the SIRI and CKD (weighted).

SIRI		Model 1		Model 2		Model 3	
		OR (95 %CI)	*P* value	OR (95% CI)	*P* value	OR (95% CI)	*P* value
**Continuous variables**							
SIRI per 1 unit		1.44 (1.38–1.51)	<0.001	1.39 (1.33–1.46)	<0.001	1.31 (1.24–1.39)	<0.001
SIRI per 1 SD		1.71 (1.58–1.84)	<0.001	1.60 (1.48–1.73)	<0.001	1.45 (1.32–1.60)	<0.001
**Categorical variable**	Events/All population						
T1 Group	1,672/6,745	Ref		Ref		Ref	
T2 Group	2,058/6,753	1.29 (1.16–1.43)	<0.001	1.30 (1.15–1.46)	<0.001	1.20 (1.04–1.38)	0.015
T3 Group	2,808/6,745	2.02 (1.83–2.23)	<0.001	1.92 (1.71–2.15)	<0.001	1.69 (1.47–1.94)	<0.001
*P* for trend			<0.001		<0.001		<0.001

Model 1: Not adjusted.

Model 2: Adjusted for age, sex, and race/ethnicity.

Model 3: Adjusted for age, sex, race/ethnicity, smoking status, alcohol consumption status, education status, the PIR, obesity status, hyperlipidemia status, DM status, CVD status, and antihypertensive drug use.

### Restricted cubic spline regression

**Figure 2.**
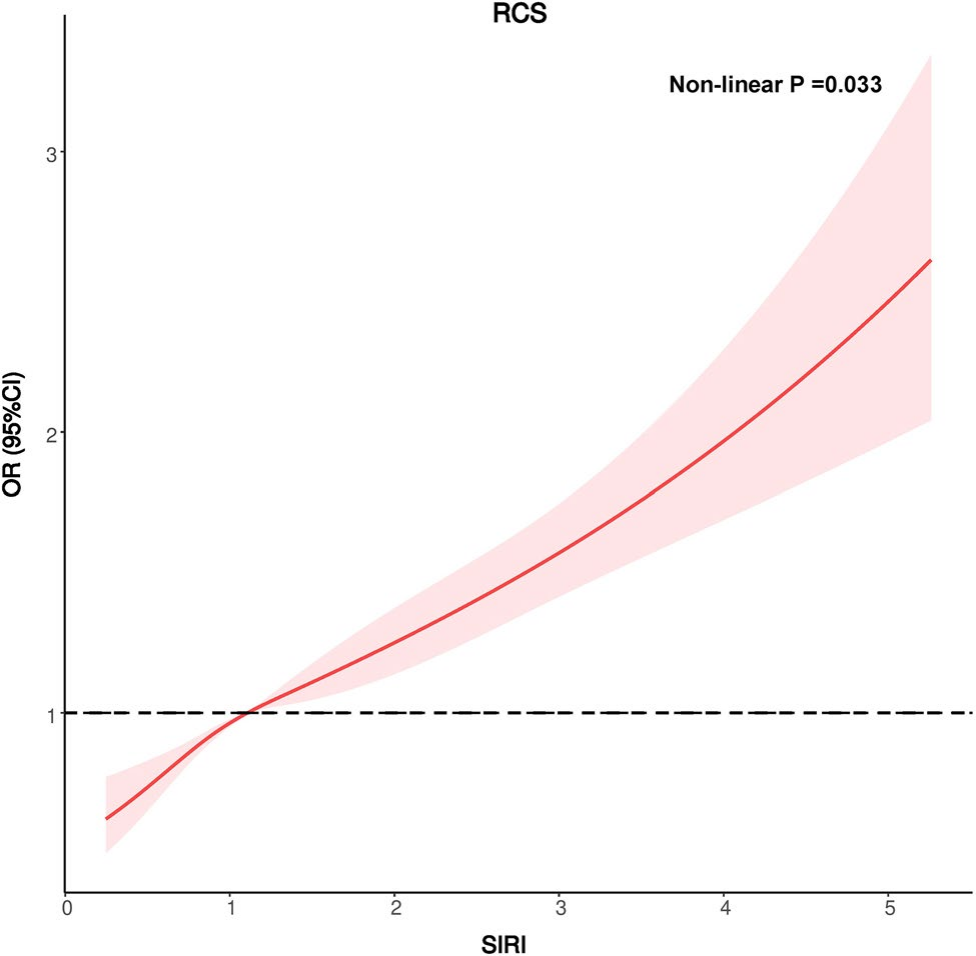
Potential nonlinear relationship between the SIRI and CKD (weighted).

In patients with hypertension, a nonlinear association (nonlinear *p* = 0.033) was observed between the SIRI and CKD based on RCS analyses. Elevated SIRIs in these patients were linked to a greater likelihood of developing CKD (Figure 2).

### Subgroup analysis

**Figure 3.**
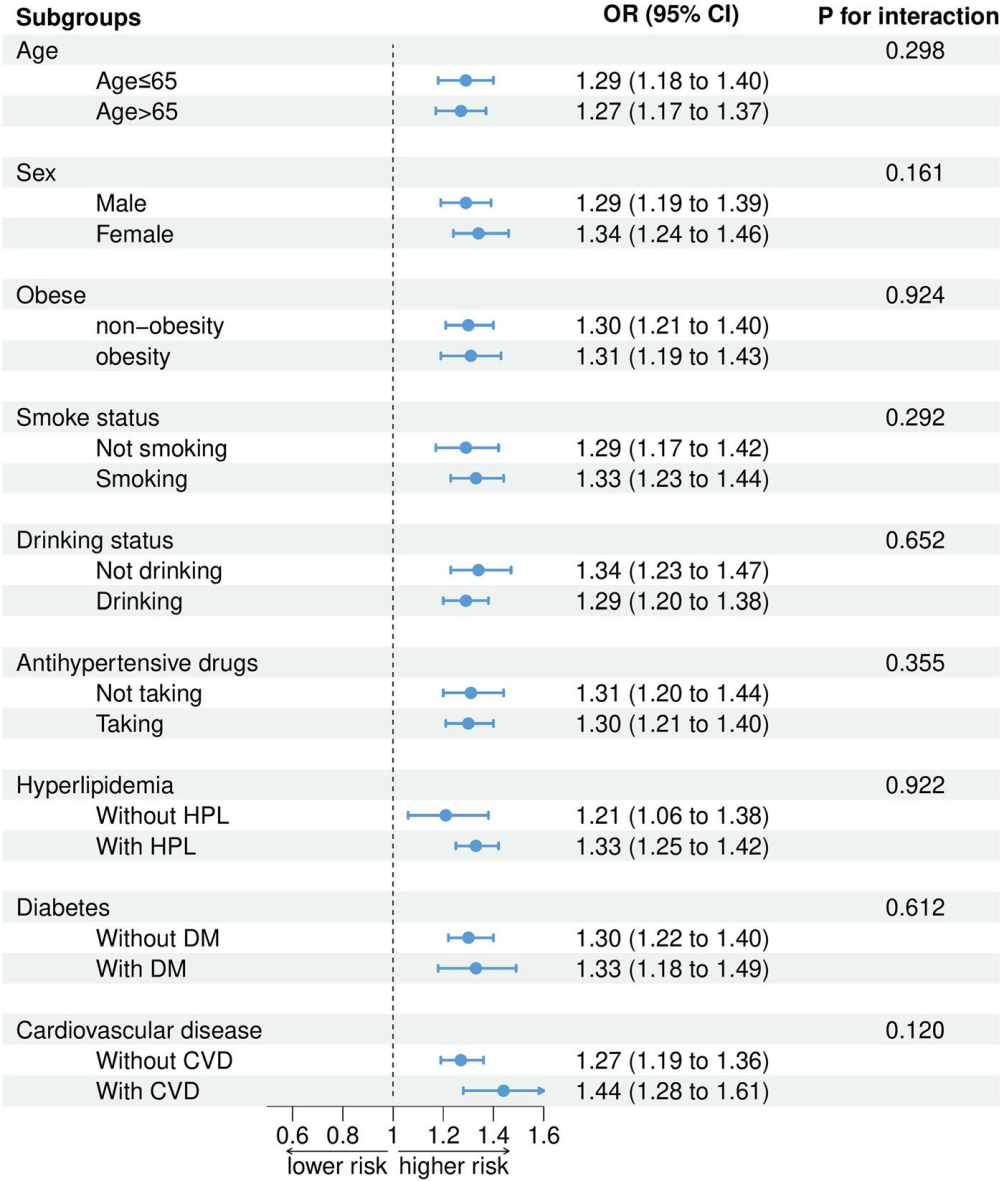
Associations between the SIRI and CKD in different subgroups (weighted).

When participants were stratified by age (*P* for interaction = 0.298), sex (*P* for interaction = 0.161), obesity status (*P* for interaction = 0.924), smoking status (*P* for interaction = 0.292), alcohol consumption status (*P* for interaction = 0.652), antihypertensive drug use (*P* for interaction = 0.355), hyperlipidemia status (*P* for interaction = 0.922), DM status (*P* for interaction = 0.612) and CVD status (*P* for interaction = 0.120), the relationship between the SIRI and the risk of CKD did not change. As the SIRI increased, so did the risk of developing CKD **(**Figure 3**).**

## Discussion

In this cross-sectional study where we utilized a representative national sample from the US, our findings indicated that the SIRI is associated with the risk of CKD in hypertensive patients. This association remains consistent across different gender, age groups, DM status, and lipid status categories.

In addition to the well-recognized press-mediated injury [19], there is a growing recognition of the link between chronic inflammation and CKD [20]. Chronic inflammation has the potential to modify and disrupt the regulation of intrarenal microcirculation and the distribution of perfusion, ultimately resulting in renal injury, the progression of renal insufficiency, and the development of chronic renal failure [21]. In addition, hypertension has been acknowledged as a disease associated with inflammation [5]. Hypertensive patients exhibit abnormal increases in inflammatory biomarkers, including tumor necrosis factor-alpha (TNF-α), as well as various cytokines and chemokines [22,23]. CKD may be influenced by the activation of these inflammatory markers *via* pathways including oxidative stress, nuclear factor-kappa-B (NF-κB), and nuclear factor E2-related factor 2 (Nrf2)[24]. In a previous study, Xu TY et al. evaluated the correlation between serum inflammatory markers and CKD in hypertensive patients and showed that the matrix metalloproteinase-9(MMP-9)/tissue inhibitor of metalloproteinase-1(TIMP-1) ratio and osteopontin (OPN) were associated with CKD in hypertensive patients [6], suggesting that the measurement of these biomarkers may provide important information about CKD in hypertensive patients. However, these biomarkers are limited by the fact that they are expensive and time-consuming to measure, making them inapplicable to large-scale community screening. Therefore, inflammatory biomarkers that can be used to screen for CKD in hypertensive patients remain to be explored.

As a novel biomarker capable of assessing inflammation throughout the body, the SIRI has been found to be associated with a variety of diseases and poor prognosis. A study conducted by Ou-Yang H et al. revealed that among people living with HIV, individuals with elevated SIRI values are more susceptible to hypertension [25]. In addition, Zhao S et al. reported that the SIRI was associated with adverse outcomes in hypertensive patients, with a greater SIRI associated with greater all-cause and cardiovascular mortality [11]. An association between the SIRI and poor prognosis has also been observed in DM patients, elderly patients with heart failure, and patients with infective endocarditis [10,26,27]. To our knowledge, there have been no investigations conducted on the associations of the SIRI with hypertension and CKD. To address this research gap, our study aimed to establish a connection between the SIRI and CKD among hypertensive patients. The findings indicated that there is an elevated risk of CKD associated with increased SIRI levels.

The theoretical basis for utilizing the SIRI to assess the risk of CKD in hypertensive patients is as follows. The SIRI is an integrated biomarker for neutrophil, monocyte and lymphocyte counts, all three of which are derived from the most basic blood parameters. As important cells of the innate immune system, monocytes can contribute to elevated blood pressure and kidney injury by releasing proinflammatory cytokines such as interleukin-6(IL-6), IL-1β and TNF-α [28]. Although the mechanism by which neutrophils are involved in hypertension has not been well demonstrated, it has been shown that higher neutrophil levels are significantly associated with the risk of developing hypertension [29,30], suggesting that neutrophils are also involved in the development of hypertension. In response to noxious stimuli, the innate immune system reacts by engaging and activating adaptive immune cells, and activated T-lymphocytes can secrete TNF -α, IL-17α, and interferon-gamma (IFN-γ), all of which also contribute to elevated blood pressure and kidney injury [28]. In addition, circulating lymphocytes are also associated with microvascular remodeling in hypertensive patients [31].

In our study, we analyzed data from 20,243 hypertensive patients and showed that those with elevated SIRI values are at high risk of CKD. Given the simplicity and ease of calculation of the SIRI, its incorporation into early screening protocols can facilitate the identification of CKD risk in hypertensive patients. There is potential benefit in initiating early intervention for hypertensive individuals with a high SIRI, as it may contribute to a reduction in the risk of developing CKD. Nevertheless, further experimental investigations are required to verify this hypothesis.

There are certain limitations to our study. First, because of the inherent limitations of cross-sectional studies, we were unable to infer a causal relationship between the SIRI and CKD, and more large-sample prospective studies are needed. Second, the SIRI was calculated from a single complete blood count rather than multiple measurements over time, which may lead to bias. Third, because our participants were all Americans, the results may not be applicable to other populations.

## Conclusion

A high SIRI is associated with an increased risk of CKD in hypertensive patients. The greater the SIRI is, the greater the risk of CKD in hypertensive patients.

## Ethics approval and consent to participate

Informed consent has been obtained from every participant and therefore there was no need for any ethical consent in this study. The NCHS ethics review board has approved the NHANES protocol. All procedures were performed in accordance with the relevant guidelines and regulations.

## Supplementary Material

Supplementary Table 1.docx

## Data Availability

All data are available as publicly accessible datasets through NHANES. It is open and publicly accessible through the following link; https://wwwn.cdc.gov/nchs/nhanes/.
