# Supplementary material for: The systemic inflammatory response index is associated with chronic kidney disease in patients with hypertension: data from the national health and nutrition examination study 1999–2018
Source: Ren Fail. 2024 Sep 23;46(2):2396459. doi: 10.1080/0886022X.2024.2396459 (PMC11421140; doi:10.1080/0886022X.2024.2396459)
Supplement: Supplementary Table 1.docx [file IRNF_A_2396459_SM0282.docx]

**Supplementary Table 1. The association between the SIR**I **and** **CKD (weighted)**

| **SIRI** | |  | Model 1 | | | | Model 2 | | | | Model 3 | | |
| --- | --- | --- | --- | --- | --- | --- | --- | --- | --- | --- | --- | --- | --- |
|  |  |  | | | OR (95% CI) | *P* value | | | OR (95% CI) | *P* value | | OR (95% CI) | *P* value |
| **Median** | Event/All population | | |  | |  | |  | |  | |  |  |
| M1 Group | 26,44/10,126 | | | Ref | |  | | Ref | |  | | Ref |  |
| M2 Group | 3,894/10,117 | | | 1.62(1.50-1.75) | | <0.001 | | 1.51(1.39-1.64) | | <0.001 | | 1.32(1.19-1.47) | <0.001 |
| **Quartile** |  | | |  | |  | |  | |  | |  |  |
| Q1 Group | 1,216/5,061 | | | Ref | |  | | Ref | |  | | Ref |  |
| Q2 Group | 1,428/5,065 | | | 1.29(1.15-1.45) | | <0.001 | | 1.33(1.17-1.52) | | <0.001 | | 1.29(1.10-1.51) | 0.002 |
| Q3 Group | 1,687/5,061 | | | 1.47(1.30-1.66) | | <0.001 | | 1.45(1.27-1.65) | | <0.001 | | 1.34(1.16-1.54) | <0.001 |
| Q4 Group | 2,207/5,056 | | | 2.34(2.09-2.63) | | <0.001 | | 2.21(1.94-2.51) | | <0.001 | | 1.92(1.65-2.23) | <0.001 |
| *P* for trend |  | | |  | | <0.001 | |  | | <0.001 | |  | <0.001 |

M1 Group: SIRI≤0.75, M2 Group: SIRI>1.11, Q1 Group: SIRI≤0.75, Q2 Group: 0.75＜SIRI≤1.11, Q3 Group: 1.11＜SIRI≤1.66, Q4 Group: SIRI>1.66

Model 1: Not adjusted.

Model 2: Adjusted for age, sex, and race/ethnicity.

Model 3: Adjusted for age, sex, race/ethnicity, smoking status, alcohol consumption status, education status, the PIR, obesity status, hyperlipidemia status, DM status, CVD status, and antihypertensive drug use.
